# Physical activity in rehabilitation practice: Policy, infrastructure and development perspectives

**DOI:** 10.4102/safp.v67i1.6137

**Published:** 2025-06-30

**Authors:** Onika Makaula, Ntandoyenkosi L. Msomi, Andrew J. Ross

**Affiliations:** 1 Department of Public Health Medicine, College of Health Sciences, University of KwaZulu-Natal, Durban, South Africa; 2 Department of Family Medicine, College of Health Sciences, University of KwaZulu-Natal, Durban, South Africa

**Keywords:** physical activity, infrastructure, rehabilitation, policy, occupational therapy, speech-language therapy, development

## Abstract

**Background:**

Integrating physical activity (PA) into rehabilitation practice is critical for promoting patient recovery and high quality of life. However, policy gaps, infrastructure constraints and resource limitations often hinder its effective implementation, particularly in public health care settings.

**Methods:**

An inductive thematic analysis of virtual individual semi-structured interviews with therapists was conducted via NVivo. Participants (*N* = 10) shared insights on their perspectives of integrating PA into rehabilitation practices regarding policy, infrastructure and development.

**Results:**

Ten subthemes emerged related to policy (two subthemes), infrastructure (four subthemes) and development (four subthemes) of PA in rehabilitation practice.

**Conclusion:**

While PA is essential for holistic patient care, therapists face systemic barriers that require policy reforms, interprofessional collaboration and investment in resources and infrastructure. Creative strategies currently mitigate these challenges but remain limited in scope of practice.

**Contribution:**

This article documents the need for policy development and resource allocation to better integrate PA into rehabilitation, whilst addressing key developmental and infrastructure gaps.

## Introduction

Physical activity (PA) is any bodily movements involving skeletal muscles that use energy expenditure^1^ and include a range of activities, such as structured exercise and activities of daily living, e.g., cooking and walking.^2^ The literature shows the essential role of PA, which ranges from preventing chronic diseases, advancing mental health and enhancing overall quality of life.^3,4^ Globally, PA is recognised as being more than the perception of mere physical fitness and regarded as essential for preventing and managing a wide range of health conditions.^5^ A study conducted in 2023 in Beijing reported that regular engagement in moderate to vigorous PA significantly reduced the risk of chronic diseases, such as cardiovascular conditions and type 2 diabetes.^4^ In addition, a study conducted in Australia in 2018 concluded that PA improves cognitive function and helps reduce the risks of cognitive decline and dementia.^6^

Piotrowska and colleagues classified PA into various categories, such as aerobic, anaerobic, balance and flexibility exercises.^7^ Aerobic exercises are activities such as cycling and walking, while anaerobic exercises include weight training and resistance exercises to build muscle strength and improve metabolic health.^8^ Balance and flexibility exercises are vital for enhancing mobility, and include yoga and tai chi.^8^ Despite the well-documented health benefits of PA, multiple challenges affect participation, specifically for those with disabilities, which include insufficient social support, a lack of access to appropriate facilities and physical limitations.^9,10^ A study conducted in South Korea in 2018 by Ji-Youn and colleagues highlighted that people with intellectual disabilities often engage in lower levels of PA compared to their non-disabled peers, which makes them susceptible to chronic conditions, such as metabolic syndrome.^11^ Furthermore, for people with disabilities, environmental factors, such as good community support, tailor made activities and availability of emotionally safe spaces, can significantly improve their participation rates, and these factors need to be addressed to promote fully inclusive PA opportunities.^12,13^

Hassett and colleagues, in their 2021 Australian study, described the relationship between disability and PA as multifaceted and complex because of inherent barriers, and these range from body structure and function to environmental and personal factors, such as self-image and perception.^14^ Furthermore, Ginis et al. emphasised that people with disabilities are less likely to meet recommended PA levels, which can exacerbate health disparities.^9^ Moreover, the severity and classification of disability determine the nature of PA participation, as seen in individuals with mobility impairments who require specialised programmes to accommodate their physical needs.^15^ Evidence-based research shows that increasing PA among individuals with disabilities can improve physical function and overall health outcomes.^10,12^ Tailored interventions that consider individual capabilities and preferences are essential for enhancing participation. For example, programmes involving family members or caregivers can foster a supportive environment that encourages PA among children with disabilities.^16^

Speech-language therapists (SLTs) are health care professionals with clinical expertise in managing communication, feeding, language, speech and swallowing-related disabilities.^17^ They are central to promoting PA, among individuals with communication disorders or disabilities, their involvement being essential for addressing speech and language impairments, and to foster overall well-being through enhanced communication, which in turn can lead to increased participation in PA. SLTs utilise various strategies to encourage active engagement in PAs, recognising the interconnectedness of communication and physical health.^17^ They promote PA by developing communication strategies that facilitate social interactions in physical settings. For instance, SLTs often train individuals with communication difficulties to express their needs and preferences effectively, which can enhance their participation in group activities, such as sports or exercise classes.^18^ By equipping people with disabilities and their families with the necessary communication tools, SLTs empower them to advocate for their involvement in PA, thereby promoting a more active lifestyle.^19^

Occupational therapists (OTs) are health care professionals with expertise in promoting PA among individuals with disabilities by facilitating engagement in meaningful activities of daily living that enhance overall well-being. Their expertise enables them to assess and modify environments, to ensure accessibility and safety, which are essential for encouraging participation in PA.^20^ OTs integrate PA into rehabilitation programmes, particularly for individuals with disabilities, by incorporating activities that promote cognitive function, motor skills and adaptive strategies into their interventions, and empowering them to engage in activities that promote social interaction and mental health.^21,22^ Physiotherapists were not included in this study to intentionally narrow the focus to OTs and SLTs, whose roles in promoting PA in rehabilitation are less well documented.

This study explores the perspectives of OTs and SLTs regarding the role of policy, infrastructure and development in integrating PA into their rehabilitation services.

## Research methods and design

### Design

A contextual, descriptive and exploratory study design was used to explore the perspectives of OTs and SLTs about the role, policy, infrastructure and development in integrating PA into rehabilitation services.

### Participants

All participants were employed in public health care facilities in the KwaZulu-Natal province. The sample included newly graduated therapists in their community service year and therapists with up to 12 years of professional experience. This combination allowed for insights from early-career therapists and those with more established clinical roles.

### Recruitment

A convenience sampling strategy was used. Poster invitations were distributed via designated Facebook and WhatsApp groups for OTs and SLTs working in public health care settings in KwaZulu-Natal. Participation was voluntary and therapists self-selected into the study based on interest and availability. While this approach enabled timely recruitment, it may have resulted in a sample biased towards individuals more invested in or aware of PA integration.

### Data collection

**FIGURE 1:**
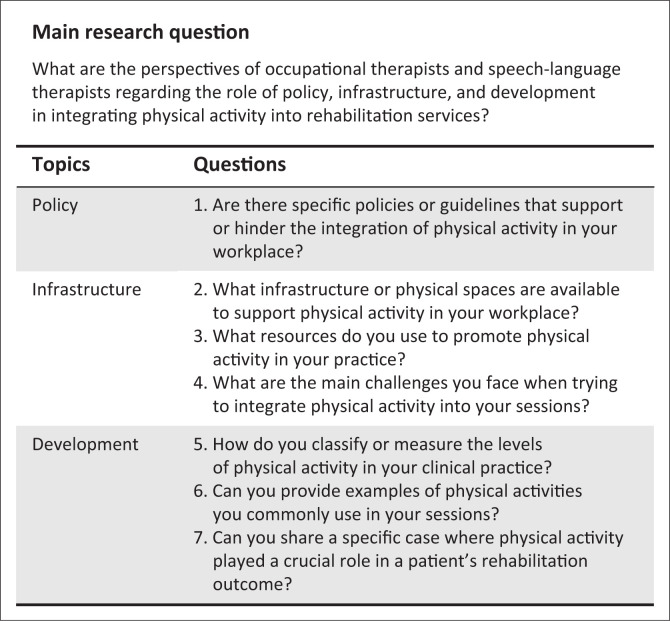
Areas or topics and their associated questions.

Individual semi-structured interviews were used to collect qualitative data from participants using the interview guide (Figure 1). The questions were designed to address the three topics of policy, infrastructure and development (Figure 1).

Data were collected virtually using Teams software, and the audio-recorded interviews lasted approximately 50 min. Interviews were conducted until data saturation was reached at 10 participants^23^ to ensure sufficient depth of the data collected and enable a robust analysis.^24^

### Data analysis

Qualitative data analysis involves condensing data to create a cohesive narrative and interpreting the findings to gain meaningful insights.^25^ Thematic analysis was conducted following a six-step process:

- *Data organisation and preparation*: The data were thoroughly reviewed and understood, including transcript reading to grasp the context.
- *Gaining a general sense of the data*: Responses were aligned with interview questions to ensure they addressed the questions posed.
- *Coding process*: Initial themes and concepts were identified by the first and second authors. Subthemes were noted and a thematic framework was applied. Themes and subthemes were refined and agreed upon by the first and second authors to ensure data trustworthiness.
- *Identifying categories or themes*: Patterns and statements were grouped based on similarities and aligned with participants’ responses and quotes.
- *Data interpretation*: Participants’ quotes were used to support the identified themes and subthemes.
- *Reporting*: The data were organised and described in detail.^26^

### Trustworthiness

This study applied the trustworthiness framework proposed by Schurink and colleagues, which encompasses credibility, transferability and dependability.^27^ Credibility was established through meaningful engagement with participants during data collection, enabling the first author to deeply understand participants’ perspectives on policy, infrastructure and development, regarding PA in rehabilitation services.^27^ Transferability was ensured by providing a detailed description of the methodology and participants’ perspectives while preserving the integrity of their responses.^27,28^ Dependability was reinforced through a two-step process in which themes and subthemes that were identified by the first author were reviewed and verified by the second author to minimise bias.^27,28^

### Ethical considerations

Ethical approval for the study was obtained from the University of KwaZulu-Natal’s Humanities and Social Sciences Research Ethics Committee on 19 July 2024 (HSSREC/00007488/2024). Participants were informed that their involvement was voluntary and that they could withdraw at any time during data collection without facing any consequences. They provided written consent and agreed to be audio-recorded during the process. All participants’ information is treated confidentially, and their identities are safeguarded. Before each interview, participants were advised not to disclose personal details, and any names mentioned during the interviews were replaced with pseudonyms to ensure anonymity.

## Results

The results section is divided into two parts: the demographic profiles and participant details are provided (Table 1), followed by a presentation of the themes and subthemes related to the topics of policy, infrastructure and development, which are supported by relevant quotes. Table 1 provides an overview of the participants’ profiles and characteristics.

**TABLE 1:** Demographic profiles of participants.

Participant code	Age (years)	Gender	Experience (years)	Profession	District
P1	23	F	< 1	OT	eThekwini
P2	23	F	< 1	OT	Harry Gwala
P3	36	F	12	SLT	Ugu
P4	23	F	< 1	OT	eThekwini
P5	23	M	< 1	SLT	eThekwini
P6	23	F	< 1	OT	uMzinyathi
P7	22	F	< 1	SLT	uThukela
P8	22	F	< 1	OT	Harry Gwala
P9	30	F	6	SLT	eThekwini
P10	26	F	3	SLT	eThekwini

OT, occupational therapist; SLT, speech-language therapist; F, female; M, male.

Table 2 indicates the themes and subthemes that emerged with respect to the three areas of interest of policies, infrastructure and development. A detailed discussion of these themes and subthemes, supported by participant quotes, is given as follows.

**TABLE 2:** Topics, themes and subthemes.

Topics	Themes	Subtheme
Policy	1. Policy and systemic barriers	1.1. Professional scope and role clarity 1.2. Need for standardised guidelines
Infrastructure	2. Infrastructure challenges in clinical practice	2.1. Limited physical space 2.2. Resource constraints
	3. Challenges in practice	3.1. Patients’ resistance 3.2. Therapist limitations
Development	4. Strategies to overcome barriers	4.1. Collaborative efforts 4.2. Innovative approaches
	5. Integration of physical activity into practice	5.1. Creative use of tools and materials 5.2. Adapting strategies to patients’ needs

### Theme 1: Policy and systemic barriers

This theme that emerged focuses on the subthemes of lack of clear guidelines, role clarity and systemic support that create challenges for therapists.

#### Subtheme 1.1: Professional scope and role clarity

Participants reported ambiguity in their roles and responsibilities, particularly about the role of physiotherapists with whom they often worked, as the overlaps sometimes created tension and hindered collaboration:

‘There’s a thing with physios, thinking we take over their job when we give exercises.’ (P1)

‘Joint sessions help define roles, but they are not always feasible.’ (P7)

We need more interprofessional collaboration to align goals. (P4)

#### Subtheme 1.2: Need for standardised guidelines

The absence of clear guidelines for integrating PA into rehabilitation was regarded as a problem, this being left to the discretion of each persons, in the absence of any fornal training on the topic:

‘How about a classification system for physical activities? There’s no standard way to measure them.’ (P8)

‘At undergrad level, we weren’t taught much about integrating physical activity, and training would help.’ (P3)

Policies should ensure access to adaptive equipment for therapists and patients. (P6)

### Theme 2: Infrastructure challenges in clinical practice

This theme addresses the limitations in physical space and resources that hinder the implementation of PA in rehabilitation settings.

#### Subtheme 2.1: Limited physical space

Participants frequently highlighted the lack of adequate physical space as a significant barrier to integrating PA into rehabilitation. Limited space constrained the variety and scope of activities that could be conducted with patients:

‘Challenge is usually one space… we didn’t normally have big spaces because of the lack of infrastructure.’ (P3)

‘If it’s raining, I can’t take the kids outside because it’s raining, and other people are also working in that same environment.’ (P9)

‘We don’t have private rooms where you can work on their stimulation.’ (P10)

These limitations often force therapists to adapt or compromise the types of physical activities provided, potentially affecting patient outcomes.

#### Subtheme 2.2: Resource constraints

Insufficient staff and inadequate resources, such as therapy materials with which to do PA, affected their ability to effictively implement PA, the therapists often resorting to improvisation to meet patient needs:

‘We don’t have balls around, so we use papers to make balls, which is not realistic for the kids.’ (P8)

‘Lack of resources in the hospital […] we use water bottles or make toys from recycled materials.’ (P2)

‘Therapy resources are very limited… sometimes we can’t do varied activities due to lack of materials.’ (P5)

### Theme 3: Challenges in practice

This theme highlights the therapists’ difficulties, the subthemes relating to patient resistance and therapist limitations.

#### Subtheme 3.1: Patients’ resistance

The participants noted that some of the problem with implementing PA arose due to resistance from patients, particularly children and those being asked to have sedentary lifestyles to address their health challenges:

‘Patients sometimes don’t see the benefits of exercises and refuse to comply.’ (P4)

‘Kids often don’t want to share toys or engage during sessions.’ (P7)

‘Patients living sedentary lifestyles lack motivation to engage in physical activity.’ (P2)

#### Subtheme 3.2: Therapist limitations

The therapists faced a number of personal challenges, including time constraints due to high patient numbers, and a lack of formal training in integrating PA. In addition the absence of senior staff to seek advice from resulting in them having to use their initiative by trial and error, and to rely on the experience and expertise of other therapists:

‘Time constraints during sessions limit the integration of physical activities.’ (P6)

‘We lack formal training on how to incorporate physical activity for complex cases.’ (P3)

### Theme 4: Strategies to overcome barriers

This theme highlights the therapists’ need to make use of collaborative efforts and innovative approaches to address challenges and enhance treatment outcomes.

#### Subtheme 4.1: Collaborative efforts

Participants emphasised the importance of collaborating with colleagues and caregivers to address challenges:

‘I prefer joint sessions with physios and OTs to align goals and avoid scope overlap.’ (P1)

‘Caregiver education is essential to ensure exercises are followed at home.’ (P4)

‘I work with caregivers to motivate patients and create a supportive environment.’ (P6)

#### Subtheme 4.2: Innovative approaches

In an effort to ensure that they provided the patients with the services they needed, the therapists employed creative solutions to resource and patient-related challenges:

‘We create toys from recyclable materials like newspapers to overcome resource shortages.’ (P2)

‘Involve caregivers in sessions to engage shy or resistant children.’ (P7)

‘Use videos on patients’ phones as reminders for home exercises.’ (P4)

### Theme 5: Integration of physical activity into practice

This theme has two subthemes that explores therapists creative use of tools and materials, and how they adapt strategies to incorporate PA into therapeutic interventions.

#### Subtheme 5.1: Creative use of tools and materials

Therapists employed innovative strategies to integrate PA into their treatment plans despite resource limitations. Participants described using everyday items creatively to simulate therapy tools:

‘We use trampolines, therapy balls, skipping ropes, and make toys like stress balls from newspapers.’ (P8)

‘I use bean bags and buckets where patients throw and name words inside the bucket.’ (P7)

‘Using five-liter water containers to simulate weights for muscle strength exercises.’ (P2)

#### Subtheme 5.2: Adapting strategies to patients’ needs

Participants tailored PA to align with individual patient needs and abilities. This adaptive approach was particularly practical for managing conditions such as cerebral palsy (CP) and attention-deficit/hyperactivity disorder (ADHD):

‘I classify activity based on the patient’s abilities, such as using low-intensity tasks for patients with CP.’ (P5)

‘For ADHD, jumping and bouncing activities help calm the patient and improve focus.’ (P8)

‘We use play-based strategies for children, like throwing a ball or kicking a ball.’ (P4)

## Discussion

There are surprisingly few empirical studies that look at the views of OTs and SLTs on PA in the context of rehabilitation. Therefore this study aimed to contribute to literature by exploring the role of development, infrastructure and policy on integrating PA into rehabilitation.

The predominance of therapists undergoing their CS year highlighted the training gaps for those without experience, and how this affects their confidence in managing their professional boundaries. Their employment in the public sector which does not have the means to provide all the infrastructure and resources required for its high patient load, is likely to have added to their concerns regarding being unable to ensure optimal services to their patients, many of whom are from poor circumstances and cannot afford to pay for items they may need for rehabilitation.

Given the growing recognition of the role played by PA in enhancing physical, cognitive and emotional well-being, it is important to understand how other rehabilitation health care professionals (SLTs and OTs) perceive its integration in rehabilitation setting. It is commonly known that PA enhances several aspects of health, such as functional ability, mental health and cardiovascular health.^29^ Engaging PA in rehabilitation settings, especially those with limited resources, can be accessible and affordable, providing a useful tool for improving patient recovery and quality of life. The study’s outcomes have greater implications for clinical practice, regulations and education. Understanding the perspectives of OTs and SLTs can help design policies that promote the inclusion of physical exercise in rehabilitation programmes. These insights help guide the creation of more all-encompassing treatment plans in the clinical setting, where PA plays a key role. The results of the study will emphasise the need for further training and resources in the field of education to provide rehabilitation health care professionals with the skills they need to be effective.

A significant obstacle to the effortless integration of PA into rehabilitation was the lack of standardised guidelines and professional role clarity, which left OTs and SLTs feeling unprepared to handle complex cases. This is consistent with findings from the study that pointed out the significance of clear professional guidelines and collaboration among professionals in improving patient outcomes.^30^ Participants also said that there was a lack of clarity on the boundaries of their tasks, particularly when they overlapped with physiotherapists, which occasionally resulted in conflicts. Even though they were acknowledged as possible answers, staffing shortages and scheduling constraints made collaborative tactics like combined sessions impractical in certain situations.

Globally, interprofessional collaboration (IPC) is acknowledged as crucial for enhancing health care outcomes. Through resource optimisation, IPC has been demonstrated to improve care quality, decrease hospital stays and increase patient safety.^31,32^ Although the benefits of IPC are widely acknowledged, systemic obstacles such as ambiguous roles, poor training and a lack of resources frequently impede its implementation. These issues are comparable to those noted in the context of physical therapy and rehabilitation, where the lack of standardised guidelines makes collaboration more difficult.^33^ Establishing common guidelines for the incorporation of PA into all rehabilitation professions including that of OTs and SLTs helps guarantee uniformity and excellence in many contexts. For example, the World Health Organization (WHO) emphasises the value of regular PA for all age groups in its extensive guidelines on sedentary behaviour and PA.^34^ These recommendations might act as a starting point for developing customised programmes that are appropriate for different patient populations.

### Infrastructure challenges in clinical practice

According to the study’s findings, PA has an important impact on patients’ rehabilitation outcomes, and needs to be included to ensure that optimal functionality is maintained. The interviewed therapists identified a number of barriers or challenges to successfully promote and integrate PA in their therapy sessions. Among those was a lack of adequate infrastructure to be used during the sessions. Participants consistently identified insufficient physical space as a major obstacle to incorporating PA into rehabilitation. The range and extent of activities that could be carried out were limited by the available space. This aligns with the findings which revealed that lack of physical space is a significant obstacle that hinders the range and depth of PAs that can be performed.^35^ Outdoor activities play a significant role in rehabilitation for SLT and OT patients. An 8-week outdoor OT programme for children with ADHD showed improvements in self-regulation, social skills, sensory processing, confidence and motor skills.^30^ Participants constantly mentioned how unavailability of enough space forced them to adapt activities, especially during adverse weather conditions or in shared environments. In addition to that, one of the participants mentioned how difficult it is to do outdoor activities when it’s raining while competing demands on the indoor spaces restricted therapy options.

Limited resources made things more difficult, so therapists had to be creative to help their patients. For instance, they formed balls out of newspapers or used water bottles as weights. It forced these professionals to use temporary fixes instead of proper equipment. For example, to improve focus and regulation, therapists used low-intensity tasks for people with CP and dynamic exercises such as leaping for children with ADHD. Play-based and tailored strategies enhance therapeutic outcomes and patient participation, especially in settings with limited resources, and this adaptability demonstrates the therapists’ dedication to patient-centred care.^36^ These methods demonstrated the inequity of the system while also demonstrating the creativity of therapists. Existing literature has also shown that not having enough resources in under-resourced areas can prevent health care workers from giving the best care possible.^37^ Workers who are strained because of a lack of resources may experience low levels of motivation and poor performance.^38^ Health care systems need to optimise make infrastructural investments, guarantee sufficient staffing and put cost-effective measures in place to improve resource allocation and utilisation to lessen these negative consequences. This entails utilising appropriate technology, streamlining processes and involving stakeholders to establish reasonable expectations and raise the standard of care generally.^38^

### Challenges in practice

The effectiveness of therapy sessions may be hampered by the difficulties that therapists frequently face, such as patient resistance and professional constraints. According to the participants, patient resistance is a widespread problem, especially with young people or those who live sedentary lifestyles. Patients may refuse to comply with PA because they do not see its benefits. These difficulties are not specific to OT and SLT as comparable obstacles are seen in different health care contexts, such as physiotherapy, where patients may be discouraged from starting or continuing treatment because of psychological issues such as anxiety and depression, a lack of motivation and fear of pain.^39^ Conversely, context-specific problems that can impact therapy outcomes include therapist limitations, such as time restraints and insufficient training in incorporating PA into their treatment plans. Participants stated that time limits during sessions restrict the integration of PAs, and a lack of trust in managing severe physical difficulties forces them to rely on joint sessions with other rehabilitation therapists. In addition, participants highlighted that they are not formally trained in integrating PA into management plans. To overcome the obstacles of patient resistance and therapist limits, training modules must concentrate on motivating for improved patient engagement and therapist confidence. Multiple studies that emphasise the value of therapist confidence and efficient communication techniques in enhancing therapy outcomes lend support to this approach.^40,41^ According to research with SLT, specialised counselling courses can greatly boost counselling confidence, which is crucial for building therapeutic relationships and enhancing patient outcomes.^42^

### Strategies to overcome barriers

To solve barriers and improve therapeutic outcomes, participants mentioned that they use creative and cooperative ways. Participants also stated that to match goals and guarantee continuous support outside of therapy sessions, cooperation with coworkers and caregivers is essential. For example, an SLT participant mentioned that to establish similar goals and prevent scope overlap, they prefer joint sessions with OTs and physiotherapists. They also mentioned that caregiver education is crucial to ensure that exercises are performed at home. Furthermore, they emphasised the significance of collaborating with caregivers to inspire patients and establish a nurturing atmosphere.

The lack of finances presented challenges with the therapists indicating that they found creative solutions to overcome them such as newspapers, which helps to meet the needs despite the resource scarcity. These collaborative efforts are consistent with recent literature on IPC, which highlights the need for teamwork to achieve better patient outcomes. For instance, IPC within clinical environments have been shown to reduce post-discharge complications.^43^ Another study found that interprofessional primary care practice models yielded significantly better clinical outcomes and highlighted how collaborative in care improves patient health outcomes.^44^

### Integration of physical activity into practice

The ability of OTs and SLTs to adjust to resource limitations shows their creativity and ability to devise solutions using the resources at hand. This flexibility is a crucial element of professional resilience, allowing them to successfully handle difficult circumstances.^45^ A study conducted in Gauteng, South Africa, demonstrated how OTs overcame resource constraints by using improvisation to make adaptive equipment for patients out of cardboard boxes and fabric scraps, illustrating the potential for innovative solutions to improve patient outcomes and engagement.^46^

Participants tailored PAs to align with individual patient needs and abilities. It was further explained in the study results that this adaptive approach was particularly practical for managing conditions such as CP and ADHD. The existing literature outlines the important role played by the patient tailored approaches in active participation and engagement of the patients in the therapy sessions, which then leads to successful therapy session with good therapist to patient relationship.^47^

In a nutshell, studies confirm the notion that innovative and flexible therapeutic approaches improve patient outcomes and engagement by strengthening the therapeutic relationship, assuring continuity of care and making interventions more approachable and relatable.^48^

### Strengths and limitations

The small sample size made it possible to collect data quickly, which is advantageous when resources or time is scarce.^49^ This made it possible for the study to respond to the research topic in a comparatively short amount of period. Considering a small sample size, the research did concentrate on getting in-depth qualitative insights from participants, which yielded rich contextual data regarding the perspectives of OTs and SLTs in KwaZulu-Natal. The use of convenience sampling poses a limitation, as the sample may not fully represent the broader population of therapists. Self-selection may have attracted participants with a particular interest in PA or innovation in clinical practice, which could introduce bias. Future studies should consider a stratified sampling method to enhance representativeness.

### Recommendations

A number of recommendations are made for the three topics as a result of this study:

1. Policy: Clear guidelines of role boundaries of these two professions against the role of physiotherapists is essential.
2. Infrastructure: Expand rehabilitation spaces to accomodate PA intervention is crucial and make provision for space sharing using a booking system to avoid scheduling conflicts.
3. Development: Include PA in higher education training of OTs and SLTs, as well as through continued professional development opportunities once they are qualified.

## Conclusion

Therapists acknowledged the benefits of PA but faced challenges such as limited space, resource shortages and unclear interprofessional boundaries. Despite these constraints, they adapted creatively to meet patient needs and deliver patient-centred care. The findings show the need for clearer guidelines, improved training and IPC. Strengthening infrastructure and policy support is essential to fully leverage the role of therapists in promoting PA as part of holistic, equitable rehabilitation services.
